# Sickness Presenteeism in Prison Officers: Risk Factors and Implications for Wellbeing and Productivity

**DOI:** 10.3390/ijerph19063389

**Published:** 2022-03-13

**Authors:** Gail Kinman, Andrew J. Clements

**Affiliations:** 1 Department of Organizational Psychology, School of Business, Economics and Informatics, Birkbeck, University of London, London WC1E 7JL, UK; 2 Aston Business School, Aston University, Birmingham B4 7ER, UK; a.clements1@aston.ac.uk

**Keywords:** presenteeism, prison officers, mental health, safety climate

## Abstract

Sickness presenteeism involves employees continuing to work while unwell. As presenteeism is influenced by contextual and individual difference factors, it is important to assess its prevalence and implications for wellbeing and productivity in different occupational groups. This study examines these issues in a sample of prison officers working in UK institutions. Data were obtained from a survey of 1956 prison officers. Measures assessed the prevalence of and reasons for presenteeism and the perceived impact on job performance, along with mental health and perceptions of workplace safety climate. More than nine respondents out of ten (92%) reported working while unwell at least sometimes, with 43% reporting that they always did so. Presenteeism frequency was significantly related to mental health symptoms, impaired job performance and a poorer workplace safety climate. The reasons provided for presenteeism explained 31% of the variance in self-reported mental health, 34% in job performance and 17% in workplace safety climate, but the pattern of predictors varied according to the outcome. The findings can be used to inform interventions at the organisational and individual levels to encourage a ‘healthier’ approach to sickness absence, with likely benefits for staff wellbeing, job performance and workplace safety climate.

## 1. Introduction

Presenteeism refers to situations where people continue to work despite feeling sufficiently unwell to take time off sick [1]. Such behaviour is commonplace, with a recent study of UK employees reporting that nearly nine out of ten (88%) had continued to attend work while experiencing illness [2] and another estimating that 1.5 working days are lost due to presenteeism for every one day lost to absenteeism [3]. Working during minor illness is not necessarily damaging and returning to work while not fully recovered via a phased return can be beneficial following more serious health problems [4]. Nonetheless, there is growing evidence that presenteeism can impair wellbeing and job performance and potentially threaten the health and safety of others [5,6]. The positive and negative implications of working while unwell are recognised in a framework developed by Karanika-Murray and Biron [7] that identifies four aspects of presenteeism: functional (working during illness without further taxing one’s wellbeing); dysfunctional (where presenteeism causes a deterioration in health and performance); over-achieving (a drive to continue working due to over-commitment) and therapeutic (enabling rehabilitation and recovery from illness).

Although in some circumstances presenteeism can be beneficial, undesirable consequences for individuals and organisations have been identified. Several reviews have highlighted the wide-ranging negative implications of working during sickness [4,5,8,9]. Longitudinal studies have found that presenteeism increases the risk of future health problems, such as depression and anxiety or coronary heart disease, as well as long-term sickness absence [10,11]. Working while unwell can also threaten job performance and lead to errors and accidents [5,12,13]. It has been argued, therefore, that presenteeism should be considered a ‘self-endangering’ and ‘risk-taking’ organisational behaviour [9,14,15] and managed accordingly. Recent evidence that presenteeism remains widespread during the COVID-19 pandemic [16,17] raises particularly serious concerns for public health, particularly when people are working while experiencing symptoms.

Research findings indicate that people work while sick for many reasons, which can be categorized as occupational, individual and organisational. Although presenteeism is found in all types of work, it is particularly common in jobs such as the ‘helping’ and ‘blue-light’ occupations, where workers typically have a strong sense of duty and a moral obligation for the welfare of others [5,17,18]. Individual difference factors such as socio-economic status and culture, personality traits such as conscientiousness, and positive orientations to work such as high engagement and perfectionism can also encourage people to work while unwell [19,20,21,22,23]. 

Organisational characteristics, such as robust attendance policies and limited entitlement to sick pay, can also increase the risk of sickness presenteeism, as can working conditions such as high demands, low control, staffing shortages, job-related stress and job insecurity [1,5,17,24,25,26,27,28]. Features of the social environment at work have also been linked to presenteeism, with a lack of support from supervisors and colleagues and experiences of bullying being key risk factors [24,29,30]. Moreover, organisational cultures can encourage presenteeism via perceptions of an ’ideal’ employee as one who shows their dedication by continuing to work through illness [1] and by stigmatisation of sickness absence where people fear being perceived as weak, lazy or inconsiderate [2]. It should be acknowledged, however, that from an employer’s perspective, sickness presenteeism may be preferable than absenteeism when staffing levels are low, especially where work is safety-critical or highly specialized [1,5].

There is evidence that sickness absence behaviours are shaped by a dynamic interplay between the characteristics of the job, the individual and the organisation [5,17,31]. It is therefore crucial to identify the factors that increase the risk of presenteeism in different occupational groups to inform the development of context-relevant interventions to reduce its damaging effects. This study focuses on prison officers, an occupational group that may be at high risk of presenteeism [32]. They work in environments where presenteeism not only has the potential to threaten their own health and safety, but can also have serious consequences for the wellbeing of others through the slips, lapses of attention and errors that can occur when people are feeling ‘below par‘. 

Prison officers work under challenging conditions in highly pressured environments. They are responsible for the wellbeing and safety of inmates in prisons that are increasingly overcrowded and understaffed and where the incidence of threats and assault is high [33,34]. Unsurprisingly, there is evidence that prison staff are at high risk of mental health problems and trauma [35,36,37,38]. Levels of sickness absence are comparatively high and frequently the result of stress-related illness, depression and anxiety, as well as recovery from physical assault [36]. In response, prisons have introduced robust attendance management policies and practices to reduce the number of working days lost [39,40]. Although careful monitoring and the threat of disciplinary action are likely to reduce short-term absence, it is recognised that the stress and anxiety they can invoke can encourage people to work when they are unwell rather than take sick leave when required [16,41]. 

A recent study of 1682 UK-based prison officers provided some insight into the extent to which they work while sick and the reasons why they do so [32]. Eighty-four percent of respondents reported working while sick at least occasionally. Officers acknowledged the negative impact of sickness absence on the safe running of prisons and recognised the need for organisational controls to discourage casual absenteeism. Nonetheless, content analysis of qualitative data highlighted several reasons that encouraged prison officers to work while sick, such as a heavy workload and robust absence management systems including ‘trigger’ systems and written warnings. Concerns about being dismissed or targeted for compulsory redundancy due to a poor sick record were also highlighted. Pressure from management to continue to work during illness or to return too soon from sick leave was also commonly thought to encourage presenteeism. 

Respondents also frequently cited a fear of letting colleagues down as a reason for presenteeism that stemmed from a strong sense of loyalty to co-workers and concerns about their wellbeing and safety, particularly if staffing was low. Concerns about being judged negatively by others and feelings of guilt and shame, as well as a strong sense of duty and professionalism, were thought to encourage people to work while sick, as low staffing levels would result in inmates being confined to their cells with little opportunity for purposeful activity or association with other prisoners. This study draws on these findings to identify the specific factors that encourage prison officers to work while sick. 

This study has several aims. Firstly, the extent to which prison officers work while unwell is examined. Secondly, the most common reasons provided for presenteeism are identified. Thirdly, as presenteeism can be a risk factor for mental wellbeing [3,4,5,17,42,43] and job performance [12,17,44] and may compromise feelings of workplace safety, relationships between working while sick and these variables are examined. Finally, the reasons for presenteeism that represent the strongest risks for mental health problems, job performance and workplace safety climate are identified. 

## 2. Materials and Methods

Sample: Data were obtained in spring 2020 from an online survey of 1956 UK-based prison officers (86% male, with a mean age of 48 (SD = 9.62)). The survey link was publicized to potential participants by a professional association that represented most prison officers in the UK. Only participants who provided complete datasets were included in the analysis. Most respondents worked on a full-time basis (93%) and the mean length of service was 19 years (SD = 10.15). The study received ethical approval and participants were assured of their anonymity and confidentiality.

Measures: In line with other studies of presenteeism [4,19] a single item was used to measure the extent to which respondents worked while sick during the previous year. Responses were obtained on a 5-point scale ranging from 1 (never) to 5 (always), with a higher score representing more frequent presenteeism. Respondents were also asked to estimate the number of days that they had worked while sick during the previous year. 

The reasons for presenteeism were assessed through a 12-item rating scale developed for the current research based on the findings of a previous study [32] of working conditions and wellbeing in prison officers. This was developed from the content analysis of qualitative data obtained from 1682 prison officers working in UK institutions. The items reflected the themes identified: heavy workload; punitive absence management systems; pressure from management; short-staffing and fear of letting colleagues down; job insecurity; concerns about disbelief and shaming; and duty and professionalism. The scale was piloted with 60 prison officers working in UK institutions prior to administration in the current study. Each potential reason for presenteeism was scored on a five-point scale where 1 = ‘strongly disagree’ and 5 = ‘strongly agree’ (Cronbach alpha = 0.84).

Mental health was measured by the General Health Questionnaire (GHQ-12), [45]. This scale is widely used to assess minor psychiatric disorders in employees. Participants indicate the frequency or severity with which they have experienced a range of symptoms, such as depression, anxiety, insomnia and impaired decision-making, compared with how they feel ‘normally’. A four-point scale ranging from ‘better than usual’ to ‘much worse/more than usual’ is used to score items. Item responses are rated from 0 to 3 and a score across items is calculated, with higher scores denoting poorer mental health (Cronbach alpha = 0.96).

Job performance was measured by the 6-item Stanford Presenteeism Scale [46] that assesses the extent to which working while sick compromises productivity. An example item is ‘At work, I was able to focus on achieving my goals despite my health problem’. Items are rated using a five-point scale where 1 = ‘strongly disagree’ and 5 = ‘strongly agree’. Higher scores denote better performance regardless of health problems (Cronbach alpha = 0.79).

Workplace safety climate was measured by a 6-item scale developed for the survey that assessed perceptions of danger and feelings of safety and security. An example item is ‘I usually feel safe on my shift’. Items are assessed on a five-point scale ranging from 1 = ‘strongly disagree’ to 5 = ‘strongly agree’. Higher scores represent more positive perceptions of the workplace safety climate (Cronbach’s alpha = 0.84).

### Analytical strategy

Descriptive statistics were calculated for each of the measures used in the study and the reasons for presenteeism endorsed by respondents. Correlations between the presenteeism scale and the outcome variables were calculated using Pearson’s *r*. Spearman’s rank order correlation was used to examine associations between the potential reasons for presenteeism and the number of days that respondents reported working while sick. Multiple linear regression was used to examine the contributions of each of the potential reasons for presenteeism in predicting the three outcome variables (mental health, job performance and safety climate). 

## 3. Results

More than nine respondents out of ten (92.3%) indicated that they attended work while unwell at least sometimes in the previous year, with 43.4% reporting that they always did so. Only 8.7% reported that they never (3.4%) or rarely (5.3%) worked while sick. Of those who reported presenteeism, the number of days worked while sick ranged from 2 to 240, with a mean of 17.08 (SD = 26.21) and a median of 10. In total, 42% of the sample reported attending work when unwell for at least 10 days, with 19% doing so for 20 days or more. 

Table 1 shows the reasons provided for presenteeism in descending order (where higher scores represent stronger endorsement). The reasons for working while sick most frequently provided were a combination of organisational and individual factors, i.e., duty and professionalism, a reluctance to let colleagues down, and feelings of guilt. Worries about job loss and reluctance to let their manager down were generally considered less important. Also shown in Table 1 are correlations between each of the potential reasons for presenteeism and the mean number of days worked while sick for respondents who reported working during illness. The strongest relationships observed were with concerns about job loss and disciplinary action as well as worries that illness would not be considered genuine, whereas the correlation with not letting one’s manager down was non-significant. 

Table 2 shows correlations between presenteeism frequency, the number of days worked while sick and self-reported mental health, job performance and workplace safety climate. Significant relationships were found between the reported frequency of presenteeism, mental health symptoms, job performance and workplace safety climate, indicating that respondents who reported working while sick more frequently tended to report poorer mental health and job performance and a more negative workplace safety climate. 

Table 3 shows the potential reasons for presenteeism and highlights the key predictors of the three outcome variables: mental health, job performance and safety climate. The key risk factors for mental health status were heavy workload, management pressure, concerns that illness was not seen as genuine, feeling guilty and concerns about job loss, with the model explaining 30% of the variance. For job performance, the most powerful predictors of poorly rated performance were heavy workload, concerns about disciplinary action, fears of disbelief and management pressure, with the model accounting for 34% of variance. Finally, the potential reasons provided for presenteeism accounted for a total of 17% of variance in perceived safety climate, with management pressure, unsafe staffing levels and heavy workload making the strongest contribution to negative perceptions.

## 4. Conclusions

This study examined the extent to which UK prison officers work while unwell, why they do so and the implications for their mental health, job performance and perceptions of the workplace safety climate. The findings confirm that presenteeism is commonplace among prison officers for several reasons that encompass aspects of the job itself, the organisation and the individual. Of some concern is that prison officers who reported working while sick typically perceived a negative impact on their job performance, a poorer safety climate at their place of work and were at greater risk of mental health problems. 

More than nine respondents out of ten reported working while sick at least sometimes and almost half indicated that they always did so. This proportion is greater than that found in a previous study of UK prison officers [32], suggesting that the pressure to engage in presenteeism may have increased over time. This is a particular concern given that the data were collected during the early stages of the COVID-19 pandemic. The scale of sickness presenteeism is alarming given that, in accordance with other research findings [9,10,11,12], working while sick has emerged as a key risk factor for mental wellbeing and job performance. Officers who worked while sick more frequently tended to report higher levels of mental health symptoms, such as depression, anxiety and insomnia, as well as cognitive impairments such as difficulties concentrating and making decisions. 

Research findings suggest that people are more likely to continue to work while experiencing mental health problems rather than physical complaints, often due to the continuing stigma surrounding such conditions [2]. Although prison officers are at a particularly high risk of poor mental health [34,35,36,37,38], they are often reluctant to disclose their difficulties to employers and to engage with support [47,48,49]. It is crucial for organisations to encourage staff to seek support at an early stage and to take sick leave if required, as continuing to work in a high-pressured and unpredictable environment may further compromise their mental health. Longitudinal studies are needed to examine the role of stigma in prison settings and how self and social stigma, such as personal beliefs, cultural norms and institutional practices, might encourage sickness presenteeism. The findings that feelings of guilt and shame and a fear of negative judgements by others were common reasons for working during illness suggest that sickness absence itself, rather than just for mental health conditions, may be stigmatised in prisons. 

This study has offered some insight into the reasons prison officers work while sick. Although the pattern of predictors varied according to the outcome, organisational demands such as a heavy workload and pressure from management emerged as risk factors for mental health, job performance and safety climate. Reflecting the stigmatisation of mental health problems discussed above, engaging in presenteeism due to fear of disbelief and feelings of guilt were among the most powerful predictors of mental health status. Working during illness because of external pressures, such as worries about being subjected to disciplinary action, and internal orientations towards the job, such as setting a good example to others, had negative implications for job performance. Continuing to work while sick due to concerns about short-staffing and letting colleagues down were among the main predictors of a poor safety climate, reflecting previous findings that loyalty, trust and mutual support are key determinants of the safety climate of organisations [50]. Previous research with prison officers has found that positive relationships between colleagues are a major source of job satisfaction that can offset the risk of work-related stressors [50]. Officers may therefore be motivated to safeguard their relationships with colleagues by working during sickness and minimize or overlook entirely the potential costs to their health and functioning. 

The implications of presenteeism for job performance were also highlighted in this study, where officers’ ability to do their job effectively was generally thought to be impaired when they were unwell. Working while sick can exacerbate the existing stressors of the job, reduce energy levels and impact negatively on task completion, especially in tasks that are more challenging. This is a major concern given that prison officers work in highly charged, safety-critical environments where feeling ‘below par’ could have serious consequences not only for their own wellbeing but that of their colleagues and the prison population as well. The short-staffing and overcrowding that is currently endemic in UK prisons and the growing risk of harassment and assault from prisoners [32,33,34] compounds these risks. 

There is evidence that interventions at the organisational level, such as reducing time pressure and increasing support, autonomy and flexibility, could potentially reduce the risk of presenteeism [26,27]. Such initiatives will also help tackle work-related stress, which is another key cause of presenteeism [17]. Organisations have a key role to play in shaping attitudes toward sickness absence. A “healthier” sickness absence culture is needed in prisons where taking sick leave is seen as responsible and considerate behavior on the part of employees and encouraged by managers. Occupational health professionals are ideally placed to advise on ‘healthy’ absence management policies and practices and work alongside employees and managers. 

Consistent, clear and equitable absence management policies and procedures are crucial in any organisation, and it is generally agreed that people who abuse sick leave should be penalised. Nonetheless, this study identified fears about disciplinary action as a key reason for presenteeism among prison officers. Reducing “unnecessary” absence without encouraging damaging presenteeism is difficult [4,5,51,52] and may be particularly challenging in safety-critical jobs where maintaining optimum staffing levels is crucial. The extent to which employees are affected by working while unwell will depend on several factors, such as the type of illness, individual reactions, the demands of the job and the available support. Where illness is minor, or provided people can work within their limits, there may be little or no risks to health or performance. The framework developed by Karanika-Murray and Biron [7] can help identify whether presenteeism is functional, therapeutic, dysfunctional or over-achieving and action can be taken accordingly. Nonetheless, raising awareness of the serious risks of presenteeism for the current and future wellbeing of staff and the safe functioning of prisons is clearly needed. It should be recognised, however, that the imperative for managers to maintain safe staffing levels in prisons could take precedence over their duty of care to their staff. To avoid future sickness absence and compromised performance over the longer-term, more staff are urgently needed and alternative ways of managing attendance among staff need to be identified.

## 5. Limitations and Future Research

This study has several limitations. The data obtained were self-reported and the design was correlational, so causality cannot be established. Further research using a longitudinal design is needed to highlight causal effects. Moreover, it is not possible to calculate a response rate as the number of participants exposed to the survey is unknown. The number of officers working in public sector prisons across the four UK nations cannot be easily established. Although the sample is substantial, it is recognised that the findings may not have captured the perceptions and experiences of the wider population. Officers who responded may have been more motivated to do so if they worked while sick more frequently or had more unfavourable opinions of their employer. The potential reasons for presenteeism assessed in the study were developed from previous research with prison officers [32] and the scale has been piloted, but further psychometric and validation work is required. Moreover, a single item was used to assess the frequency of presenteeism. While this approach is commonplace [4], future studies should use multi-item measures to identify the prevalence of presenteeism among prison officers working in different settings, the type of symptoms or diseases most linked to working while sick in such environments and their implications for health and job performance. For example, prison officers are vulnerable to sleep disorders which can lead to on-the-job fatigue that can compromise their safety [53], whereas physical health conditions are likely to limit their ability to assist colleagues during critical incidents. This study provides some evidence that sickness absence among staff is stigmatised in prisons, but future research might identify the types of illness that are considered legitimate and illegitimate causes for taking time off sick. Insight is also needed into managers’ and officers’ beliefs about how working while sick impacts on job performance in a safety-critical, highly interdependent working environment. The data used in this study were collected before the COVID-19 pandemic. It is likely that organisational norms about working while sick will have been influenced by the pandemic [17], where continuing to work while sick, especially with an infectious disease, may be more stigmatised than presenteeism. Finally, socio-economic and demographic variables, such as salary and terms of employment, are likely to influence the frequency of presenteeism and the reasons for such behaviour. This should be examined in future research. 

## Figures and Tables

**Table 1:** Reasons for presenteeism in descending order (higher scores represent higher levels of endorsement) with descriptive statistics and correlation coefficients with mean presenteeism days.

Item	Mean	SD	*r_s_*
Duty and professionalism	4.29	0.87	0.06 ***
Not letting colleagues down	4.07	0.99	0.16 ***
Feeling guilty	3.87	1.19	0.17 ***
Worried about disciplinary action	3.68	1.29	0.23 ***
Unsafe staffing levels	3.60	1.19	0.17 ***
Expectations of other people	3.49	1.13	0.18 ***
Pressure from management	3.46	1.28	0.21 ***
Concerns illness not seen as genuine	3.44	1.27	0.20 ***
Setting a good example	3.33	1.15	0.06 *
Heavy workload	3.31	1.17	0.14 ***
Worried about job loss	3.17	1.35	0.27 ***
Not letting manager down	2.91	1.21	−0.00

The range for each of the presenteeism items was 1–5. *** *p* < 0.001; *p < 0.05.

**Table 2:** Correlations between study variables.

Study Variables	*M*	*SD*	1	2	3	4	5
1. Presenteeism	3.30	0.59	1.0				
2. Days worked	17.08	26.21	0.15 ***	1.0			
3. GHQ-12	1.50	0.81	0.28 ***	0.31 ***	1.0		
4. Performance	2.90	0.74	−0.13 ***	−0.24 ***	−0.58 ***	1.0	
5. Safety climate	4.71	0.74	−0.25 ***	−0.17 ***	−0.45 ***	0.31 ***	1.0

*** *p* < 0.001.

**Table 3:** Predictors of outcome variables: mental health, job performance and safety climate.

Reason for Presenteeism	Mental Health β	Job Performance β	Safety Climate β
Worried about disciplinary action	0.02	−0.16 ***	0.06
Worried about job loss	0.12 ***	−0.07 *	0.04
Pressure from management	0.16 ***	−0.13 ***	−0.27 ***
Unsafe staffing levels	0.06*	−0.08 **	−0.19 ***
Not letting colleagues down	0.04	−0.03	−0.13
Not letting manager down	0.05 *	−0.11 ***	−0.10 ***
Concerns illness not seen as genuine	0.14 ***	−0.15 ***	0.01
Feeling guilty	0.13 ***	−0.12 ***	0.01
Duty and professionalism	0.09 **	−0.12 ***	0.02
Expectations of other people	0.11 ***	−0.11 ***	−0.08 *
Heavy workload	0.18 ***	−0.21 ***	−0.11 ***
Setting a good example	0.02	−0.12 ***	0.03
Total R_2_	0.30 ***	0.34 ***	0.17 ***

*** *p* < 0.001; ** *p* < 0.01; *p < 0.05.
